# Supplementary material for: NMR Profiling of Ononis diffusa Identifies Cytotoxic Compounds against Cetuximab-Resistant Colon Cancer Cell Lines
Source: Molecules. 2021 May 28;26(11):3266. doi: 10.3390/molecules26113266 (PMC8198399; doi:10.3390/molecules26113266)
Supplement: Supplementary file 1 [file molecules-26-03266-s001.zip › molecules-1218279-supplementary.pdf]

NMR profiling of *Ononis diffusa* identifies cytotoxic compounds against cetuximab-resistant colon cancer cell lines

Vittoria Graziani [1,2], Nicoletta Potenza [1], Brigida D'Abrosca [1], Teresa Troiani [3], Stefania Napolitano [3], Antonio Fiorentino [1,*] and Monica Scognamiglio [1,*]

SUPPLEMENTARY MATERIAL

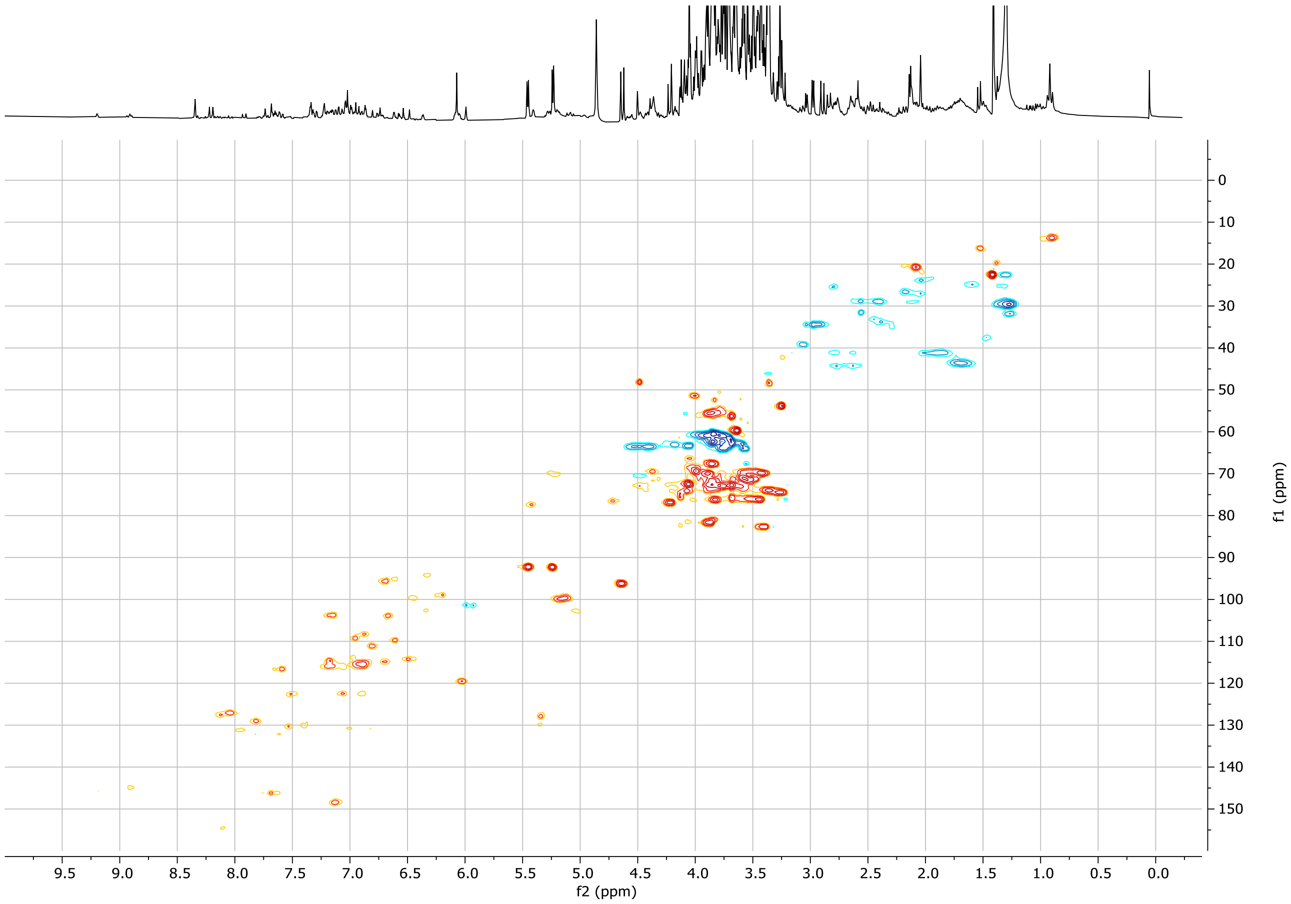

Figure S1. HSQC of *O. diffusa* extract

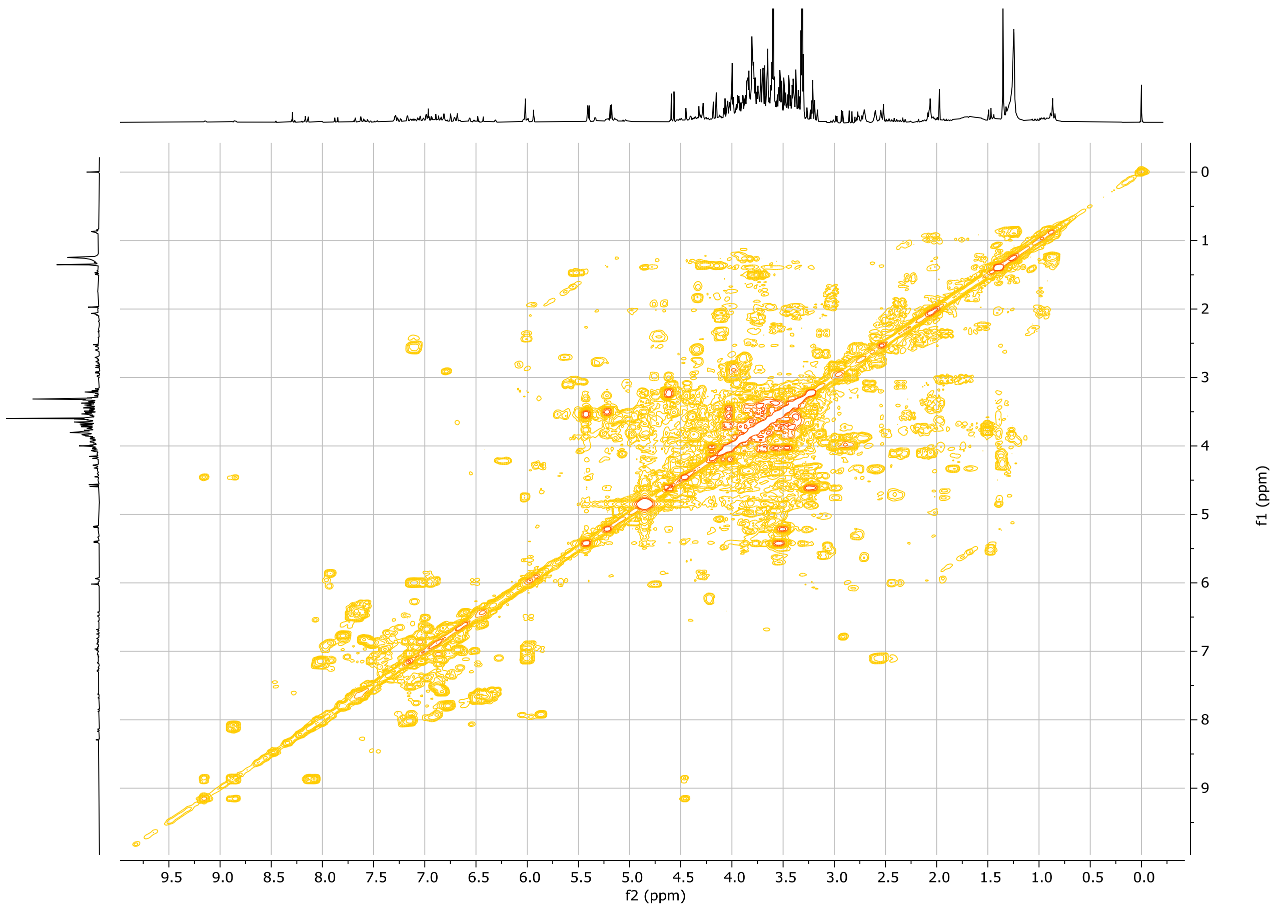

Figure S2. COSY of *O. diffusa* extract

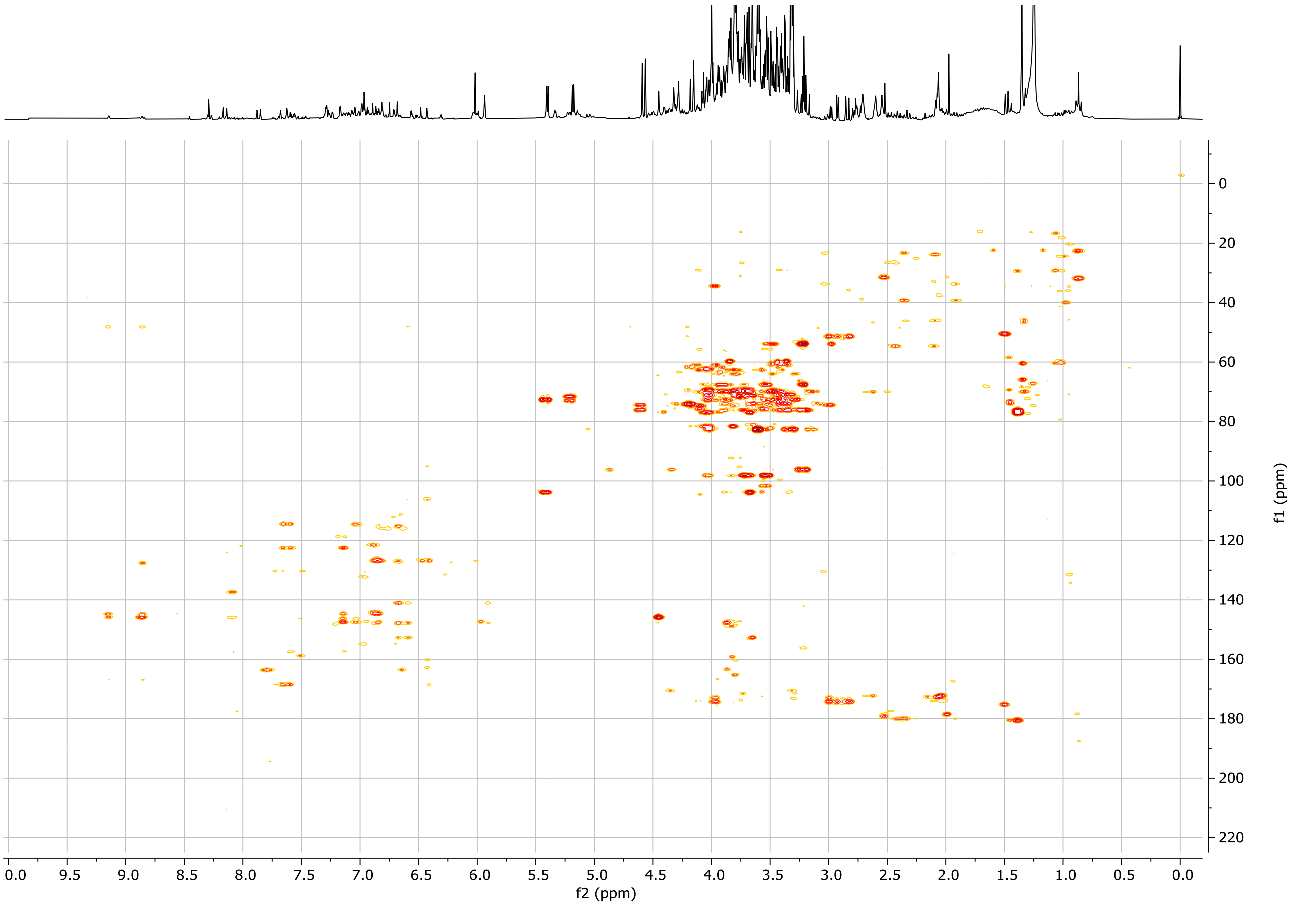

Figure S3. HMBC of *O. diffusa* extract

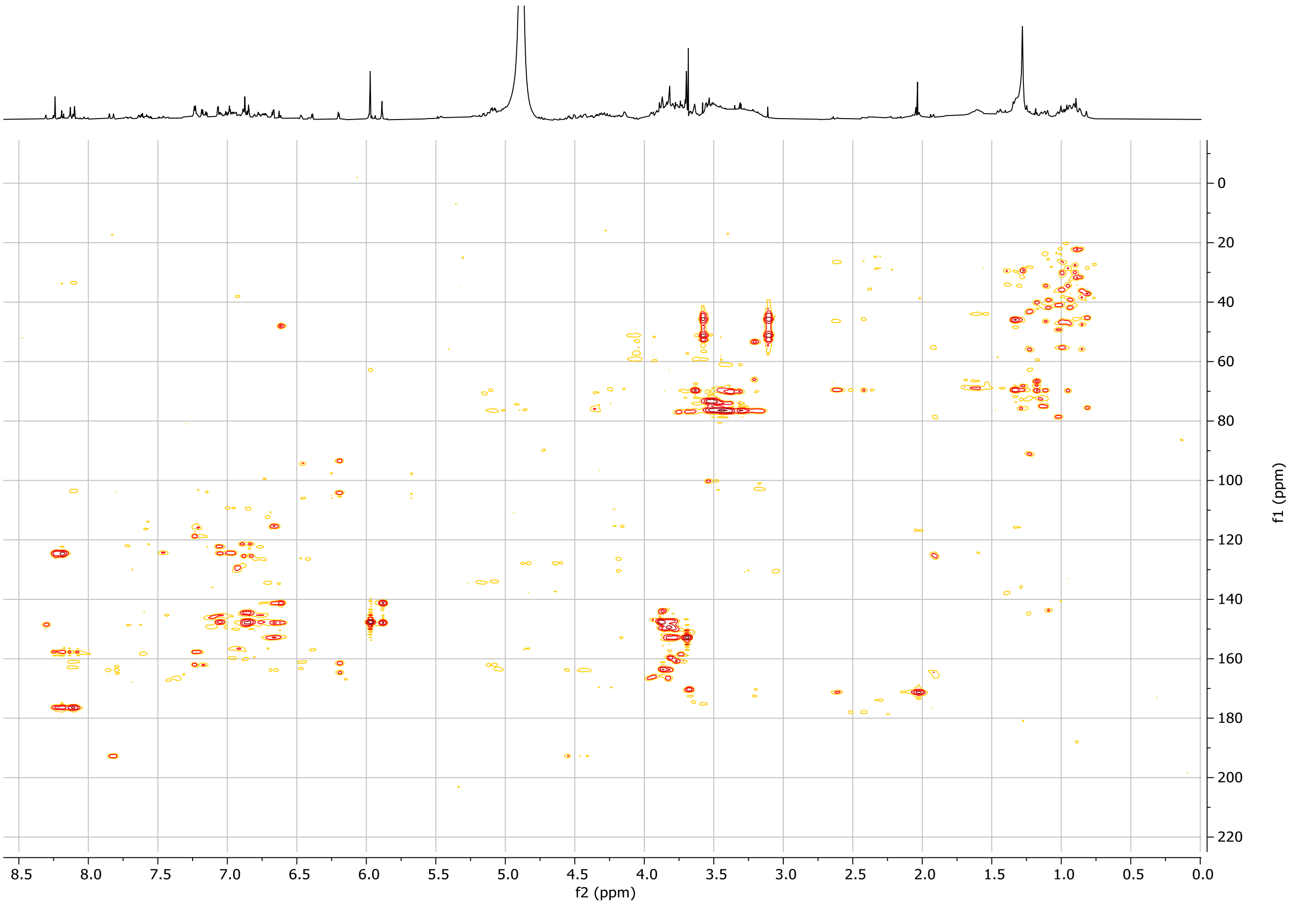

Figure S4. CIGAR-HMBC of OdM fraction

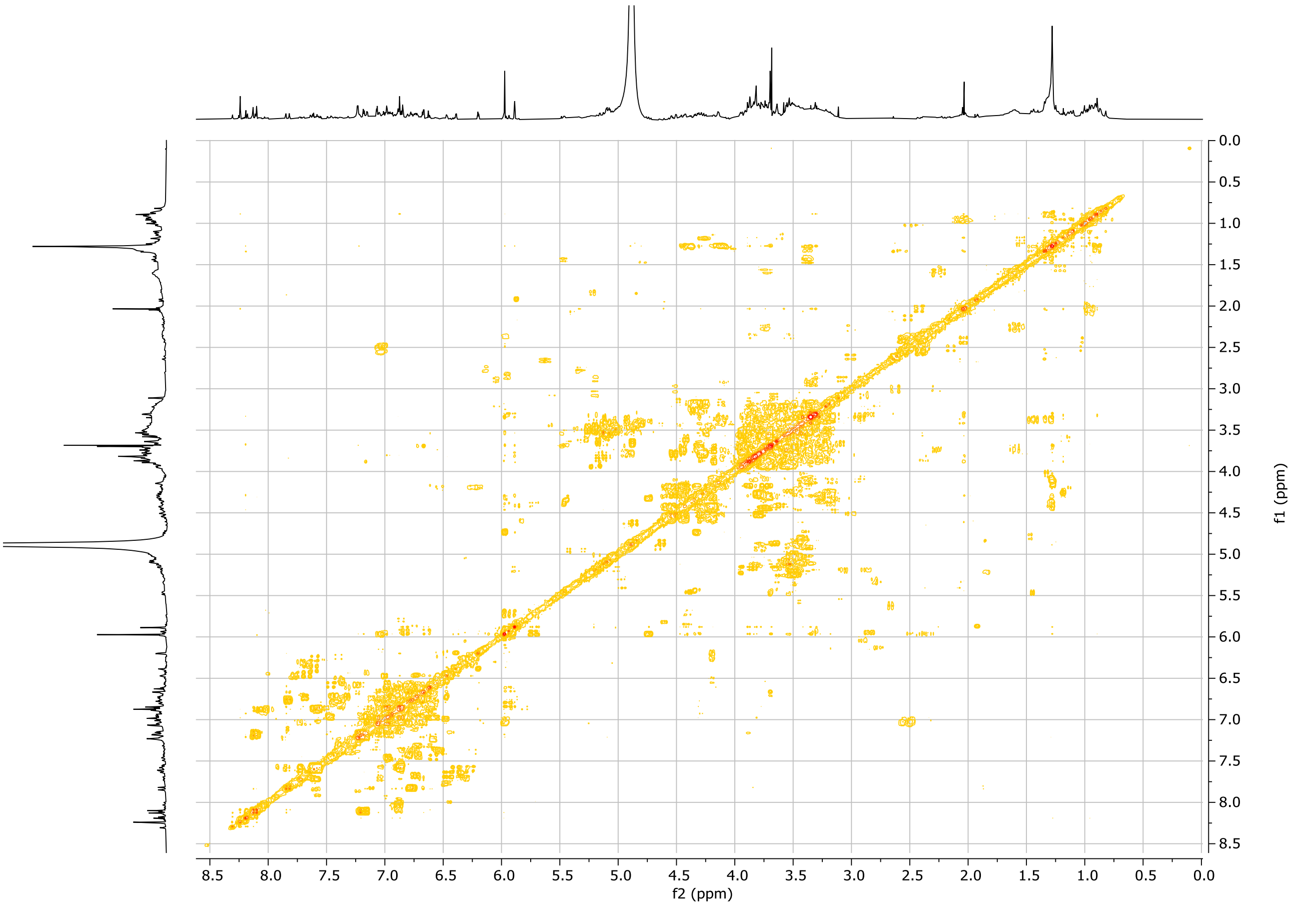

Figure S5. COSY of OdM fraction
